# A qualitative exploration of young adult smokers’ responses to novel tobacco warnings

**DOI:** 10.1186/1471-2458-13-609

**Published:** 2013-06-25

**Authors:** Janet Hoek, Anna Hoek-Sims, Philip Gendall

**Affiliations:** 1 University of Otago, P O Box 56, Dunedin, New Zealand

**Keywords:** Smoking, Young adults, Warning messages, Temporal construal

## Abstract

**Background:**

Despite reduced smoking among adolescents, smoking prevalence peaks among young adults aged 18–30, many of whom believe themselves exempt from the health risks of smoking shown in warning labels. We explored how young adult smokers perceived warnings featuring proximal risks, and whether these encouraged cessation more effectively than traditional health messages.

**Methods:**

We conducted in-depth interviews with 17 young adult smokers and explored their perceptions of current warnings as well as novel warnings representing short-term health consequences; immediate social risks, and tobacco’s toxicity (denormalizing tobacco as an everyday product). We used a thematic analysis approach to explore how participants rationalized existing warnings and interpreted the novel messages.

**Results:**

Participants considered the immediate social and physiological benefits they gained from smoking outweighed the distal risks shown in health warnings, which they regarded as improbable and irrelevant. Of the novel warnings, those presenting immediate social risks altered the balance of gains and losses young adults associated with smoking; however, those presenting short-term health risks or depicting tobacco as a toxin were less effective.

**Conclusions:**

Participants regarded warnings featuring proximal social risks as more salient and they were less likely to rationalise these as irrelevant. Social risk messages merit further investigation to examine their potential as a complement to traditional health warnings.

## Background

Because smoking causes millions of preventable deaths each year governments have used many interventions to deter smoking initiation and prompt cessation [1-7]. These have included social marketing and industry denormalisation campaigns, increasing the excise tax on tobacco products, greatly limiting marketing, providing subsidized nicotine replacement therapies, and introducing smoke free work and recreation areas [8-10].

Despite these measures, tobacco packaging, which uses imagery and brand names to elicit specific and very desirable connotations, remains an important marketing medium [11-14]. Symbolic consumption theory suggests consumers use brands to access aspirational attributes such as glamour, coolness, femininity or ruggedness assert their social identity, and publically demonstrate their group membership [15-23]. Tobacco brand associations thus undermine tobacco control initiatives by reassuring smokers, impeding their quit attempts, and deflecting attention away from health warnings [24,25].

Pictorial warning labels (PWLs) disrupt brand symbolism by presenting alternative messages and challenging the connotations branding creates [26]. PWLs have reduced tobacco packaging’s appeal [3], increased awareness of the harms attributable to smoking, stimulated message processing, and elicited higher levels of cessation-linked behaviours than text-only warnings [27,28]. Because smoking causes fatal and debilitating illnesses, health warnings aim to increase knowledge of the widespread harms directly attributable to smoking and prompt cessation. However, while these messages resonate with older smokers, many of whom have experienced symptoms of illness, they may have less effect on younger smokers, who reportedly see the messages as lacking relevance and realism [29]. This lack of salience enables young adult smokers to employ self-exempting strategies that diminish the risks shown, thus rationalising and supporting their continued smoking [30,31]. As smoking prevalence peaks among this demographic [32], there is an urgent need to develop warnings young adults see as salient, and that deter initiation, reduce progression to addiction. and encourage cessation [23,33].

Research from the United States suggests denormalizing strategies that challenge perceptions of tobacco as a ‘normal’ product might counter the images of glamour and rebelliousness young people associate with smoking [34,35]. Chapman and Freeman suggested denormalization would spoil smoking’s social acceptability, undermine its desirable connotations, and expose it as no more than a toxic behaviour [36]. Denormalization reduces the immediate benefits young adult smokers receive from smoking, questions the social persona they construct using brand imagery, and repositions smoking not as cool, but passé. These messages do not try to persuade young adults they risk serious health problems in later life, but instead undermine the immediate social and psychological benefits they hope to access by smoking [37].

Temporal construal and prospect theory provide a framework for exploring denormalization further; both challenge ‘rational choice’ models of behaviour and suggest people respond differently to the same event, depending on whether they associate it with losses or gains [38] (p.265). Whether individuals believe a decision will produce positive or negative outcomes depends on how the decision is framed and, crucially, on when they might experience the perceived consequences [39,40]. Individuals’ construal of an event depends on its proximity [41]; people view events in the long term future as more abstract and uncertain relative to proximal events, which they see more clearly and assess with greater certainty [42]. Proximal rewards, available in the near future, offer more value than distal, or deferred, rewards. As a result, aversive consequences perceived as more distant exert less influence than those that manifest more quickly [41,43].

Smokers receive near-instant physiological rewards from smoking as well as potential peer approval, while the asserted benefits of cessation - a promise they might live longer and die of something other than a disease caused by smoking - are deferred [40,44]. Consequently, these benefits lack salience when compared to the discomfort caused by unsatiated nicotine cravings, as well as the possible embarrassment that may result if smokers decline to smoke when those around them do [40].

As forgoing smoking or quitting might have direct negative consequences, while continuing has few immediate disadvantages, young adult smokers have little incentive to heed warnings they regard as conditional and so distal as to be irrelevant. Messages that challenge smokers’ temporal perspectives might be more likely to affect how they assess the gains and losses of smoking, and their ability to rely on self-exempting beliefs [45]. Although Oakes *et al.* found older smokers were more likely to express self-exempting beliefs [46], recent work concluded that adolescents may be even more likely to display self-exempting behaviours [47]. Because rationalising smoking’s risks enables smokers to distance themselves from dissonance-inducing health consequences, self-exemption reduces motivation to quit and has prompted calls for work examining how self-exempting rationalisations could be challenged.

Temporal construal theory suggests that focussing less on distal health consequences and more on proximal social effects could challenge young adults’ self-exempting beliefs, thus altering how they assess the gains and losses of smoking [48,49]. This approach may also elicit stronger emotional responses, which several studies have linked to increased message effectiveness [50,51].

To date, however, denormalization messages that challenge smokers’ temporal perspectives have not been translated into on-pack warnings. Nor have denormalization messages been tested in countries where the tobacco industry has a lower profile and wields less political influence. Using data from in-depth interviews, we explored how New Zealand young adult smokers interpret current tobacco warning messages and investigated their response to novel messages that use denormalization approaches to reframe the proximity of smoking’s risks.

New Zealand represents a unique tobacco control context as the government has set a goal of the country becoming a smokefree society by 2025 (defined as smoking prevalence below five percent) [52]. Currently, 17% of New Zealanders smoke, though prevalence varies greatly by age and ethnicity; it rises among young adults, of whom around 30% smoke, and peaks among young Māori women, of whom around 50% smoke [32]. The need for increasingly innovative measures to achieve New Zealand’s 2025 goal may have wider international relevance as more countries establish similar ‘end game” objectives [53].

In our research we explored the following research questions:

RQ1: How do young adult smokers interpret and assess the risks they face from smoking?

RQ2: How do young adult smokers interpret health and social warnings regarding smoking?

## Methods

The New Zealand Health Research Council (HRC) funded the study. Ethical review was undertaken by a delegated authority from the University of Otago’s Human Ethics Committee. Participants reviewed a detailed information sheet and received an oral summary of their key rights, including the right to withdraw from the research at any time, ask a question at any time, and decline to answer any question. Each participant had an opportunity to ask questions or seek clarification before giving written consent to participate in the research. To ensure participants’ confidentiality, we assigned each a pseudonym and de-coupled their transcript from any identifying information.

We collected data in 17 depth interviews with young adult smokers (eight women and nine men) aged 18 to 30 who were self-defined social or daily smokers. In-depth interviews allow more detailed probing than is typically possible in a focus group and we used this opportunity to undertake a careful exploration of participants’ perceptions and rationalisations.

To achieve a diverse sample that included participants from varied backgrounds, we recruited participants purposively using local advertising (posters displayed in cafes, libraries, supermarkets and welfare offices). We also made direct approaches to people in the relevant age range observed smoking (these approaches aimed to increase sample diversity) and used social media (Facebook) to elicit participants whose age, gender and ethnicity varied. Interviews took place in two New Zealand cities (one large - population >100,000 - and one provincial - population <100,000) during January and February 2011. All participants were offered gift vouchers valued at $30 (these included phone cards or book tokens, neither of which could be redeemed for tobacco) to recognize their assistance. Table 1 contains details of participants’ demographic characteristics and smoking behaviours.

**Table 1:** Participants’ characteristics

	**Name (pseudonym)**	**Gender**	**Age**	**Education**	**Smoking status (self-assessed)**	**Average cigarettes/day**^**1**^
1	Alex	Male	20	No formal qualification	Daily	10-15
2	Belinda	Female	18	School qualification	Daily	5+
3	Cam	Male	28	Bachelor’s degree	Social	1-3
4	Debby	Female	24	School qualification	Daily	10+
5	Eddy	Male	20	School qualification	Social	1-2
6	Felix	Male	21	Bachelor’s degree	Daily	5
7	Gary	Male	23	No formal qualification	Daily	~10
8	Hannah	Female	20	School qualification	Social	<1
9	Izzy	Female	21	Certificate or diploma	Social	2-5
10	Jan	Female	24	Certificate or diploma	Daily	~10
11	Katie	Female	28	School qualification	Social	1-2
12	Lloyd	Male	23	School qualification	Daily	~10
13	Mike	Male	21	Certificate or diploma	Social	1-2
14	Nick	Male	21	School qualification	Daily	5
15	Owen	Male	21	School qualification	Social	2-3
16	Penny	Female	28	School qualification	Daily	8-10
17	Queena	Female	21	School qualification	Social	2-3

^1^Several participants gave ranges for the number of cigarettes they smoke each day and noted they smoked more when with friends, particularly when drinking. Social smokers averaged their daily consumption.

The interview protocol was loosely structured and comprised primarily open-ended questions that included introductory, follow-up, probing, specifying and indirect questions [54]. We first explored participants’ smoking initiation and subsequent smoking trajectory, and their awareness and perceptions of current warning labels. Participants then viewed the test messages (presented randomly), explained their interpretation of the images, and commented on how these made them feel about smoking. Interviews took place in several venues (homes or private meeting spaces in public libraries) according to participants’ preferences, and ranged from 35 to 50 minutes in length. With participants’ permission we recorded the interviews, which were then transcribed, reviewed and analyzed for themes. Two researchers conducted each interview, reviewed the recordings, and agreed that data saturation (assessed as no new idea elements) had occurred following fifteen interviews. We conducted a further two interviews as an additional check that saturation had occurred.

**Figure 1:**
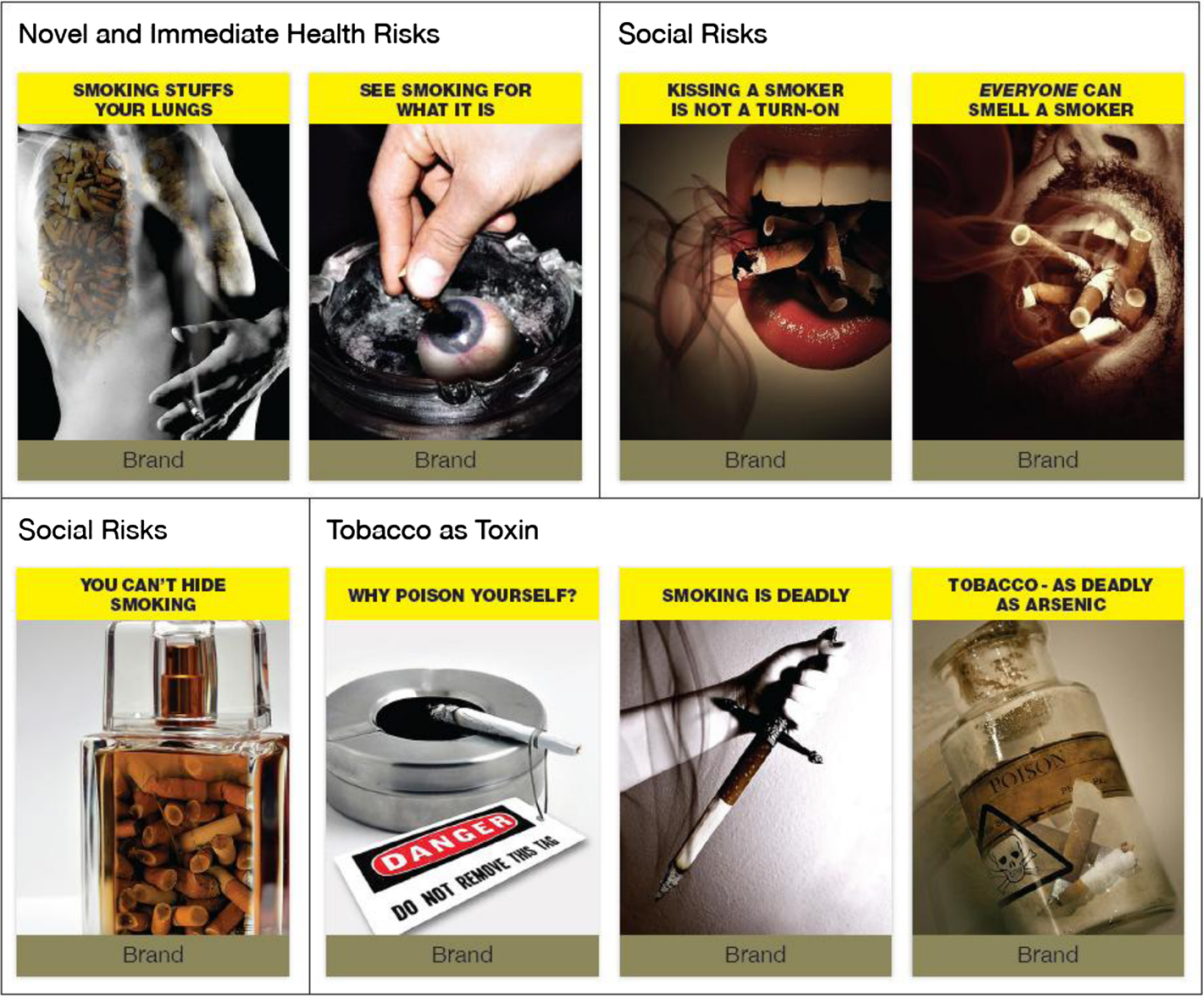
Test warning messages.

A graphic artist developed warning images that reframed the immediacy of smoking’s risks and drew on denormalization themes identified in the literature; the taglines used evolved following discussion between the researchers and the artist, and the resulting images underwent an informal peer review with graphic design students. Figure 1 contains the test messages; two presented health risks in novel manner to challenge beliefs that smoking harms only heavy, long-term smokers. Young adult smokers do experience loss of fitness and women, in particular, report experiencing badly bloodshot eyes following binge smoking (and drinking) sessions [55]. The reframed health risks focused on these two proximal outcomes of smoking. Because smokers feel self-conscious about the smell of smoking, three images denormalized smoking as a social behaviour by highlighting the overt smell and taste of smoking, and the difficulty of disguising its pungent and unpleasant odour. The remaining three images denormalized tobacco as an everyday product by recasting it as toxic and deadly, akin to a poison that would cause immediate harm. Reframing smoking in this way challenged relegation of smoking’s harms to distal (and irrelevant) points on smokers’ personal timelines and repositioned risks as more immediate.

Following transcription, we analysed responses to each message theme using thematic analysis [56]; the two lead authors independently read (and re-read) the transcripts to ensure familiarity with the content and identify similarities and differences in participants’ comments. On the third reading, we identified idea elements and used an inductive approach to develop preliminary themes in relation to the three denormalization approaches. We then compared and reached agreement on these themes [57] before checking them against the research questions and wider literature.

## Results

We first explored participants’ response to current pictorial health warnings (PHWs) and identified one overarching theme: participants’ use of time in their rationalization and rejection of warning messages. In response to the test warnings we identified three themes: invulnerability to health risks; social ostracism, and recognition of tobacco as a toxin. Quotations are annotated to indicate participants’ gender (F, M) and age.

### Time as a rationalizing tool

Temporal construal theory suggests young adults regard distal risks as less certain, and thus less salient. Participants’ comment illustrated how their perceptions of time affected their responses to existing warnings: “*It* [the warning] *doesn’t really change anything … it’s just a picture, it hasn’t happened to me yet*” (F, 18). Evidence nothing had happened “yet” suggests participants disregard distal and conditional outcomes and consider these irrelevant to their current behaviour. When probed, these comments revealed an underlying belief that smokers controlled their smoking and would quit when they chose: “*We’ve known for years that this isn’t good for us, so I’m just going to do it until I don’t want to*” (F, 21). Ironically, the participant uses the fact we have “*known* [about tobacco’s harms] *for years*” to assert her control over smoking and minimize any immediate risk she might face. She is not ignorant of the risks, but defies their relevance until she is ready to quit.

Many participants distanced the effects of smoking to absolve themselves of risk, which they believed applied only to older, long-term smokers. Even if they found PHWs unsettling, participants resolved their discomfort by arguing their youth made them invulnerable to the problems shown: “*it kind of like goes off in my head, like a warning sign, but… I’m like too young now, so kind of carefree* (laughs), *I guess… I’m not going to care about this for at least twenty or thirty years. You now, I’m not at risk at the moment of whatever kind of thing*” (F, 21).

Even when they obliquely considered the prospect of becoming a long-term smoker, participants did not confront smoking’s risks but instead used exceptions to reinforce their self-exemptions and challenge the veracity of the images. Thus anecdotal evidence from a friend proved more compelling than medical evidence, and examples that questioned health risks had greater perceived credibility than scientific findings: “*I heard this from my friend who says her mum has been smoking her entire life and she is still alive and I think has no cancer, no disease, but people who don’t smoke get lung cancer*” (M, 28). Participants used claims that non-smokers may also suffer from lung cancer to diminish the risk smoking posed to them. Evidence others had defied time by smoking for an “*entire life*” allowed participants to view smoking as presenting no greater risk than “*anything”*, to which we all are vulnerable: “*I guess I just know so many people that smoke for years – years and years – and never got sick or anything. Well, I might not, but you know anything can kill you these days*” (F, 24).

Only a minority discussed feeling unsettled by current PHWs and did not use time to dispel risks: “*I should never have done that* [smoked]… *I should have just left it. You know, I should have just not agreed with it at all and just not smoked*” (M, 21). For this participant, regret has reduced his temporal perspective; he looks backwards to a point he wishes he had avoided, rather than forward to a future he believes he can control.

Overall, while most participants claimed they felt unaffected by current warnings, they diminished the threats presented either by interpreting the messages as too distal to be relevant or by using ad hoc, time-resistant, examples to challenge their validity. They reframed smoking’s consequences as distal possibilities rather than immediate certainties and drew on exceptions to distance themselves from the inevitability of smoking’s harms.

The test images participants viewed attempted to challenge these self-exempting beliefs by reframing risk as a more proximal outcome. Specifically, warnings presented more immediate health consequences, highlighted smoking’s social unacceptability, and recast smoking as a poison that could inflict immediate harm.

### Invulnerability to health risks

Participants’ rationalizations of current warnings deferred smoking’s consequences and enabled them to rest secure in their belief they will quit before they faced a ‘real’ risk. The novel health-oriented warnings aimed to highlight proximal outcomes by focusing on problems young smokers experience (bloodshot eyes and loss of fitness). Male participants, in particular, found the stuffed lung image easy to understand because they empathized with the experience depicted: “*I usually play soccer, I like sport, and I can feel like my lungs are not as good as before*” and “*I know it’s bad for your lungs, because I’ve noticed that*” (M, 28). Because the warning highlighted a salient problem for these participants, they did not regard the consequences as deferred, but understood them as real and immediate. Recognition that they were ‘stuffing’ their lungs (literally and metaphorically) translated into fear for some: “*It makes a pretty big impact because it kind of shows the lungs filling up…* [and made me feel] *may be a little bit scared of what’s going to happen in the future*” (M, 21). Nevertheless, most participants differentiated between the symptoms shown, which they found inconvenient, and risk, which they continued to regard as a distal outcome.

However, participants found the second image, which depicted unattractive bloodshot eyes (an immediate consequence of binge drinking and smoking and a common practice among young adults) harder to understand. Participants commented: *“It just, yeah, you know, doesn’t really mean a lot to me (laugh)”* (f, 28)*.* Nevertheless, a small group recognised the multiple messages the image communicated: “*It* [smoking] *is dangerous, that it is harmful,… it’s not fair on people who don’t smoke, it’s not fair on your families as well, who might think you shouldn’t smoke… you see that it costs you a lot of money. There are a lot of things that can come up from that statement and it does make you think about them. I don’t think everyone would realize that though*” (F, 24). Paradoxically, although participants rationalized the risks they faced from smoking, they felt unable to dismiss these risks on others’ behalf and the thought they might inflict harm or pain on others created an unsettling tension.

Overall, participants had strong aversive reactions to the two images reframing smoking’s health risks as more immediate and salient; they found the images graphic and uncomfortable, and most disliked looking at them. Yet, even though they recognized the symptoms depicted, most projected serious risks to the future and saw proximal risks, such as loss of fitness or sore, bloodshot eyes as immediate, but manageable. As a result, while participants acknowledged smoking is harmful, few reported experiencing personal concern or felt they were more likely to make a quit attempt in the near future. Instead, many relied on a general belief that they would quit smoking at some undefined future time, before which they thought it unlikely they would face any health risks.

### The risk of social ostracism

Many smokers belong to social groups with high smoking prevalence; smoking thus represents a badge of group membership and contributes to smokers’ social identity. Several participants reported starting smoking to share a group norm: *“It’s just everybody did it, so all my friends did it…”* (F, 18) and noted they had started *“social smoking, in the beginning, then gradually moved into smoking most days”* (M, 21)*.* Nevertheless, the fact that smoking has become increasingly socially unacceptable means smokers risk social alienation when they venture beyond these groups. Several participants had experienced external disapproval and reported modifying their behaviour to avoid others’ judgments:

“P: It’s not really that socially acceptable anymore… I don’t smoke in public, I only smoke at my house.

I: And why is that?

*P: Um, I dunno, it’s kind of embarrassing.”* (M, 23)

Three images reflected the paradox that smoking simultaneously symbolises group identity and yet is socially alienating by presenting smoking as an unsocial behaviour defined by its strong and aversive smell. Participants found images of men and woman smokers’ mouths easy to interpret, relevant, and unpleasant: “*it’s staying that if you are going to smoke, you may as well chew the cigarette because you’re going to smell* (F, 28) and “*It looks really gross with the mouth full of butts. I can’t imagine that would be very pleasant, like using your mouth as an ashtray, which I guess, to a degree, smokers do*” (M, 21).

The mouth images tapped into participants’ fears and experiences, and elicited self-conscious responses from both men and women. One recognised the loss of physical appeal affecting smokers: “*I’d feel pretty yuck kissing someone who smokes if that person doesn’t*” (M, 20), while several commented on the distasteful smell of smoking: “*Yeah, it does actually stink. I’ve gone through a weekend when I hadn’t had any, and I couldn’t stand being around people who did smoke because you could smell it”* (M, 23). These participants reported being acutely aware of smoking’s smell, which they found unappealing: “*I’m always quite conscious about the smell of smoke and stuff because I don’t like it … yeah, that one definitely works for me because it’s something that I’m quite conscious of… the dirtiness of it all*” (M, 21).

Because smoking has a distinctive smell, participants felt exposed to others’ judgment, which they experienced as ‘shaming’. The mouth images reframed smoking as a behaviour that led not to social approbation, but to rejection: “*It’s not cool smelling like a cigarette all the time and people who don’t smoke smell you, they are like, yeah, I don’t know, give you a look or something… it’s pretty shaming*” (F, 18). Rather than face being rebuffed by non-smokers, smokers took steps to appease those whose disapproval they feared: “*I reckon that definitely the smell’s terrible… I’m always conscious of it… I always stay outside an extra five minutes to try and get rid of some of the odour before I go inside… I’m always trying to put spray or cologne, or take breath mints… for other people’s comfort*” (M, 23). The warnings confronted participants with smoking’s immediate negative consequences, which they recognized as familiar problems that already troubled them and limited their social encounters.

Smoking’s unavoidable smell, and others’ reaction against this, led some participants to describe smoking as a socially rejected behaviour that lost its aspirational attributes: “*It’s not hot and cool as it may have been back before everyone knew the hazards and definitely brings to mind that whole kissing an ashtray kind of analogy*” (F, 24). Participants dealt with this dismantling of smoking’s desired qualities in two ways. For some, the knowledge brought regret, because their addiction linked them to a behaviour that potentially threatened their social acceptability. However, a minority changed their temporal perspective to discount the discomfort they felt. They held strongly to beliefs they would become smokefree in the future: “*it doesn’t really change my opinion on smoking… I don’t plan on being a lifelong smoker, but I know that happens… these pictures and things, they don’t really change my opinion on smoking itself*” (M, 21). Temporal inversion legitimized this participant’s behaviour: because he would not always smell or taste potentially offensive to others, he could manage the short-term problem represented in the warnings.

Participants also found the perfume bottle image easy to understand: “*It’s like the picture, to me it looks like it’s true because you can’t really hide anything. You can’t hide smoking*” (F, 24). Many empathised with the message: *“It stinks. Like it really does and no matter what you try and do, like, if you have a smoke, then you’re just going to smell of smoke.... It’s a pretty nasty smell.”* (M, 21)*.* However, although several reported having tried unsuccessfully to disguise smoke’s distinctive odour, this image elicited more reactance than the other social risk images. Daily smokers, in particular, reported feeling untroubled by the message, particularly if they typically associated with other smokers: *“most people that smoke a lot of cigarettes anyway are in a comfortable environment where they don’t have to hide it”* (M, 20). Participants entrenched in a smoking network recognised that others may feel repelled by the smell of smoke, but rarely had to confront this problem: *“I do realise it’s a really potent stench, smoking, it’s like wrong that you can smell it. Um, but I’m surrounded by lots of people who do smoke… so the idea of smoking is quite comfortable, there’s not someone kind of saying, criticising, you know, feeling comfortable, so I’ve never really had that need to realise…”* (F, 24)*.* The inconsistency between participants’ everyday experiences and the message presented meant the image lacked salience and impact, and was more easily discounted.

Overall, social smokers found the mouth warnings particularly effective, largely because they did not feel at risk of the health problems caused by smoking: “*I think kissing a smoker, especially for young people, relationships and being successful in socialising and what not with the opposite sex or you know. I think it’s quite important for young people*” (M, 23). Some thought smokers would be aware of the health warnings, but may not have fully considered the social risks they ran: “*So they’re not trying to tell you smoking is harmful and deadly – you might have heard that before – but these are actually new things that people might not have heard before*” (F, 24). Although most participants were sensitive to the smell of smoking, messages heightening this sensitivity had high cut-through and impact, and participants saw them as a more salient and effective cessation trigger than health-oriented warnings.

### Tobacco as toxin

The final message theme denormalized smoking’s status as ‘cool’ and aspirational; the warnings presented tobacco as a toxin that could inflict immediate harm. Participants understood the poison and self-harm messages easily, felt unsettled by these, and found they reduced the experience of smoking: “*It makes you feel kind of shifty about smoking; every time you have a cigarette you think about it, like poisons and stuff, rather than just enjoy it*” (M, 21). For some participants, the images connoted attributes they had relegated to the distant future and reconnected smoking with outcomes they wished to avoid: “*it’s a menacing kind of object, it means a lot of things, harmful things like death and stuff like that*” (F, 24).

Yet depicting tobacco as a poison and smoking as akin to self-harm stimulated counter-argument and rationalization; several participants debated the time that needed to elapse before harm could occur and placed more emphasis on their absence of overt symptoms than on future risk: “*The thing is, you know, having one smoke isn’t, might not, isn’t going to kill you in the short term. People will look at that and be like, oh smoking, you know, I’m going to smoke this smoke, but I’m not going to drop dead after i*t” (M, 23). This participant rationalized his behaviour by disaggregating it; smoking could not have harmful cumulative effects because each individual act had no short term consequences.

As young adult smokers do not experience immediate negative effects after smoking, they discounted the message content: “*Saying it’s poisoning you and that you’re going to get heart disease, like it doesn’t really have an effect on me because I don’t have any of those symptoms, so I don’t think I’ve going to*” (F, 24). This participant’s rationalization again draws on time; the lack of proximal effects supported her extrapolation that she would not experience harm in the future and enabled her to dismiss the message. Participants also projected the consequences, a strategy that reduced the need for immediate behaviour change: “*It’s not really deadly, as such, only after a long period of time*” (M, 23). Overall, the toxin and harm images generally had weaker visual impact and failed to challenge smokers’ lived experiences, where the immediate consequences of smoking were not harmful, but beneficial and reinforcing.

Table 2 summarises participants’ responses to the message themes and the images used to illustrate these. Overall, the health and ‘mouth’ images had the strongest visual impact, although only the latter were consistently easily understood, salient, and represented an immediate threat.

**Table 2:** Summary of participants’ responses to warnings

**Image**	**Summary evaluations**
Novel Health Themes	
Stuffed Lungs	• High visual impact;
	• Easily understood;
	• Strong salience (especially with male participants);
	• Risk seen as distal and inconvenient rather than life-threatening
Eyeball	• High visual impact;
	• Poorly understood;
	• Limited salience, though multiple messages resonated with the few who understood these;
	• Not seen as presenting an immediate risk.
Social Risk Themes	
Male and female mouth (two images)	• High visual impact;
	• Easily understood;
	• Strong salience with both males and females;
	• Immediate risk that stimulated concern despite reported precautions
Perfume bottle	• Weaker visual impact;
	• Not easily understood;
	• Strong salience with both males and females;
	• Immediate risk but lower levels of concern
Product Denormalisation Themes	
Dagger	• Moderate visual impact;
	• Easily understood;
	• Weak salience;
	• Not seen as presenting an immediate risk.
Poison bottle	• Weaker visual impact;
	• Easily understood;
	• Weak salience;
	• Not seen as presenting an immediate risk.
Ashtray	• Weaker visual impact;
	• Easily understood;
	• Weak salience;
	• Not seen as presenting an immediate risk.

## Discussion

Our findings are highly congruent with earlier studies that document young people’s perceptions that health warnings featuring long-term risks lack salience and relevance [29,48,49]. To our knowledge, this is the first study to use temporal construal theory to inform interventions designed to address young adult smokers’ risk self-exempting practices. Evidence of temporal manipulation suggests self-exempting beliefs about smoking’s risks may be countered by highlighting proximal risks; further research is now required to estimate how temporally-adjusted risk messages affect likely cessation behaviours.

Of the novel warnings tested, the health and social risk images typically had the strongest visual impact. However, messages highlighting smoking’s social unacceptability and smokers’ loss of attractiveness had stronger salience and immediacy, proved less amenable to temporal relocation, and also merit further investigation. These messages reduced smoking’s symbolic value, tainted the identity participants sought [10,25], and foregrounded the risk that non-smokers would reject them as unattractive and undesirable [36,37].

Social smokers found these messages especially salient and felt challenged by images that suggested smoking may lead to social isolation rather than acceptance [44]. Reframing a known risk in a novel manner generated high immediacy and elicited fewer dismissive rationalizations. Although earlier work suggests social smokers rarely regard themselves as smokers [28,34,58], and so exempt themselves from health risks, our participants could not avoid identifying with social warnings, which they considered highly relevant to all young people. Associating smoking with off-putting and unpleasant smells spoiled connotations of glamour or rebellion, tainted its symbolic attributes, and reminded smokers of the dissonance smoking induced in them [26,36,59].

However, identifying more proximal risks did not always promote greater message acceptance among participants. An image of a lung physically and metaphorically ‘stuffed’ from smoking reflected experiences participants recognized, reminded them they were already experiencing negative outcomes of smoking, but fell short of representing a serious and behaviour-changing risk. Similarly, while salient, a more abstract message featuring a bloodshot eye and urging smokers to ‘see smoking for what it is’, had less effect, even though participants disliked the image. A likely explanation for this lack of impact is that the image itself was too metaphoric and left participants unclear about its meaning. Despite depicting proximal health risks, participants saw these as inconvenient, yet manageable, because they believe they will quit before facing any health risks. Participants had similar responses to images that reframed tobacco as an immediate poison rather than an everyday product. These images also had little effect and either lacked visual impact (poison bottle and ashtray) or did not present an immediate risk (all images); they thus fell short of providing the impetus needed to stimulate a quit attempt.

Because we probed smokers’ understanding and interpretation of novel stimuli, the study concluded once data saturation had occurred and the overall sample size is small. Future work is now required to test whether our participants’ perceptions reflect wider population responses, particularly in respect of cessation-related behaviours, such as quitting. Such work could assess the relative effects of health and social warnings, particularly their effects on general risk beliefs and short-term risk perceptions, examine how different executions function, and estimate how distinct negative emotions, such as fear and disgust, affect cessation intentions. While earlier studies analysed the effects and ethics of fear-arousing messages [60,61], disgust has received more recent attention [62-64] and merits further scrutiny. We know little about how disgust-arousal affects feelings of personal vulnerability, or whether it stimulates cessation-related responses, elicits reactance, or has other effects.

As well as examining alternative message themes, future work could explore responses following multiple exposures to novel messages and assess the relationship between novelty and message salience. Longitudinal qualitative work, used in other tobacco control studies, has potential to identify changes in temporal perspectives and the triggers linked to these, and could inform future message development [65,66]. Such work could also examine whether reactance promotes stronger quit intentions and more frequent quit attempts [67].

Assuming replication and extension studies such as these corroborate our findings, processes for implementation will require investigation. In some countries, such as New Zealand, current legislation could allow greater warning message diversity. However, other jurisdictions may require revision of existing regulations or new legislation. As many countries have indicated their desire to introduce plain packaging, legislation introducing this measure could also include provisions enabling use of more diverse warning messages, where these have a robust evidence base.

## Conclusions

This study extends our understanding of how young adult smokers interpret, understand and rationalize smoke-free stimuli. In particular, the warnings presenting social risks undermined the identity participants sought and the benefits they hope to derive from smoking, and suggest temporal relocation of risk outcomes has potential to challenge young adult smokers’ self-exempting beliefs. Social smokers were more responsive than daily smokers to messages that challenged their temporal perspectives, particularly those focussing on smoking’s unpleasant smell. These messages elicited less rationalizing and rejection than health or poison messages, and were more likely to stimulate cessation-related thoughts. Nevertheless, given some participants’ difficulty in interpreting more metaphoric messages, further work is required to test alternative executions and assess whether message novelty, immediacy or salience is the best determinant of effectiveness.

As thousands of young people experiment with smoking each year and go on to become addicted smokers, findings that offer richer insights into the style and content of smoke-free messages have potentially important public health implications. Because smoking prevalence peaks among young adults, it is timely to examine whether more diverse warnings could deter smoking initiation and prompt cessation more effectively among the demographic that would benefit most from quitting. This study offers directions for future work that could assist policy makers, health researchers and social marketers to develop more effective messages for the diverse sub-groups that exist among smokers.

## Competing interests

We have no financial or non-financial competing interests. However, for the sake of full disclosure we note that this study was funded by the Health Research Council of New Zealand (grant 09/195R). JH has received funding for tobacco control research from the Royal Society of New Zealand (Marsden Fund) and the Cancer Society of New Zealand. PG and JH have received funding for tobacco control research from the New Zealand Ministry of Health and the Heart Foundation of New Zealand.

## Authors’ contributions

JH conceived of the study and with AH-S and PG designed and pre-tested the protocol. AH-S and JH collected and analysed the data. JH led the MS development. AH-S and PG contributed to the final MS. All authors have read and approved the final MS.

## Pre-publication history

The pre-publication history for this paper can be accessed here:

http://www.biomedcentral.com/1471-2458/13/609/prepub
